# Non-Invasive Alcohol Concentration Measurement Using a Spectroscopic Module: Outlook for the Development of a Drunk Driving Prevention System

**DOI:** 10.3390/s24072252

**Published:** 2024-04-01

**Authors:** Yechan Cho, Wonjune Lee, Heock Sin, Suseong Oh, Kyo Chang Choi, Jae-Hoon Jun

**Affiliations:** 1 Department of Biomedical Engineering, Konkuk University, Chungju-si 27478, Chungcheongbuk-do, Republic of Korea; zhdnrk05@kku.ac.kr (Y.C.); ckdqh501@naver.com (W.L.); gh7486@gmail.com (H.S.); susung233@gmail.com (S.O.); 2 Road Innovation Technology, Jincheon-gun 27856, Chungcheongbuk-do, Republic of Korea; lionseven@hanmail.net; 3 Research Institute of Biomedical Engineering, Konkuk University, Chungju-si 27478, Chungcheongbuk-do, Republic of Korea

**Keywords:** alcohol concentration measurement, non-invasive sensing, spectroscopy, biosensor

## Abstract

Alcohol acts as a central nervous system depressant and falls under the category of psychoactive drugs. It has the potential to impair vital bodily functions, including cognitive alertness, muscle coordination, and induce fatigue. Taking the wheel after consuming alcohol can lead to delayed responses in emergency situations and increases the likelihood of collisions with obstacles or suddenly appearing objects. Statistically, drivers under the influence of alcohol are seven times more likely to cause accidents compared to sober individuals. Various techniques and methods for alcohol measurement have been developed. The widely used breathalyzer, which requires direct contact with the mouth, raises concerns about hygiene. Methods like chromatography require skilled examiners, while semiconductor sensors exhibit instability in sensitivity over measurement time and has a short lifespan, posing structural challenges. Non-dispersive infrared analyzers face structural limitations, and in-vehicle air detection methods are susceptible to external influences, necessitating periodic calibration. Despite existing research and technologies, there remain several limitations, including sensitivity to external factors such as temperature, humidity, hygiene consideration, and the requirement for periodic calibration. Hence, there is a demand for a novel technology that can address these shortcomings. This study delved into the near-infrared wavelength range to investigate optimal wavelengths for non-invasively measuring blood alcohol concentration. Furthermore, we conducted an analysis of the optical characteristics of biological substances, integrated these data into a mathematical model, and demonstrated that alcohol concentration can be accurately sensed using the first-order modeling equation at the optimal wavelength. The goal is to minimize user infection and hygiene issues through a non-destructive and non-invasive method, while applying a compact spectrometer sensor suitable for button-type ignition devices in vehicles. Anticipated applications of this study encompass diverse industrial sectors, including the development of non-invasive ignition button-based alcohol prevention systems, surgeon’s alcohol consumption status in the operating room, screening heavy equipment operators for alcohol use, and detecting alcohol use in close proximity to hazardous machinery within factories.

## 1. Introduction

Alcohol is a component found in alcoholic beverages and is one of the various central nervous system depressants, acting as a psychoactive substance [1]. If one engages in drunk driving, it can lead to issues such as impaired visual function, decreased driving abilities, and cognitive impairments [2]. This poses a significant problem for safely operating a vehicle. According to the World Health Organization’s (WHO) Global Road Safety Status Report, it is possible to ascertain the global rates of alcohol-related road traffic fatalities. The official percentages of alcohol-related road deaths in various countries in the year 2010 ranged from 2% to 38% of total road traffic fatalities [3]. Alcohol is a substance that can impair functions such as the brain, cognition, and muscle control [4,5]. Driving under the influence of alcohol can lead to accidents due to factors such as fatigue and impaired cognitive attention, resulting in collisions with obstacles or inadequate responses to emergencies [6,7]. Drivers under the influence of alcohol are seven times more likely to cause fatal accidents than regular drivers [8]. Many countries around the world are experiencing social and economic losses due to drunk driving [9].

According to the National Highway Traffic Safety Administration (NHTSA) in the United States, approximately 37 people die in alcohol-related traffic accidents every day, which translates to roughly one person every 39 min [10]. In 2021, there were 13,384 fatalities in alcohol-related driving accidents, showing a 14% increase compared to the previous year, 2020. Another issue with drunk driving accidents is the high likelihood of repeat offenses [11,12,13]. Among individuals with prior convictions for drunk driving, the recidivism rate increased by 13.5% within two years. Furthermore, regardless of whether the blood alcohol concentration (BAC) was high or low, both categories saw an increase in recidivism rates.

In order to address these issues, each country enacts laws and conducts alcohol enforcement using alcohol measurement devices. The most common breathalyzer, which is used for alcohol measurement, poses hygiene concerns due to the need for a mouthpiece during testing [14]. Invasive measurement methods involving blood sampling are highly accurate but time-consuming for concentration determination, making immediate detection of alcohol consumption difficult [15]. There exists a method of alcohol measurement through the detection of air within the vehicle [16]. However, this method has the drawback of low accuracy as it can also detect substances containing alcohol, such as mouth freshener or vehicle fragrance. In addition to this alcohol measurement technique, various other methods have been researched. Electrochemical alcohol measurement utilizes changes in electromotive force due to oxidation reduction reactions within an electrolyte [17]. However, this method requires periodic calibration, is susceptible to external factors like temperature and humidity, and can experience device fatigue with repeated use. Gas chromatography involves injecting the target bodily fluid into a separation column [18]. Substances are separated by heat and enter individual detectors. The concentration of alcohol is determined based on the amplitude of detector responses. However, this method is costly, experimentally complex, not suitable for continuous measurements, and requires operator expertise. Semiconductor-based measurement sensors measure changes in conductivity due to adsorption and catalytic reaction [19,20]. Nevertheless, these sensors exhibit non-specific responses to alcohol, reduced sensitivity over time, and a limited sensor lifespan as measurement time progresses. Enzyme-based alcohol concentration measurement methods utilize dehydrogenase enzymes to convert ethanol into acetaldehyde and reduced NADH [21]. The reduced NADH, which is proportional to the ethanol concentration, is measured using optical methods. However, this method has a limited range of alcohol concentration and limited accuracy. PCF (Photonic Crystal Fiber) alcohol sensing exhibits high measurement sensitivity due to significant changes in the optical refractive index but is influenced by temperature, affecting both refractive index and measurement sensitivity [22]. OECT (Organic Electrochemical Transistor)-based enzyme reaction alcohol sensors can be produced quickly and inexpensively but require continuous replacement as they are disposable devices [23]. Patch-based alcohol concentration measurement methods detect alcohol metabolites in sweat to sense alcohol concentration [24]. However, the accuracy of alcohol concentration is low due to the influence of sweat, and the periodic replacement of the patch is necessary due to limited usage time. 

A research paper was published using a custom-built NIR interferometer to measure alcohol concentration on the forearm [25]. In the research, a fiber optic array was employed as the probe, and an integrating sphere was utilized for data collection. Subsequently, the same research group used a commercially available product, TTT-1100, to measure alcohol concentration on the forearm using a similar method [26]. Although the mentioned paper conducted some in vivo studies on the forearm, it is essential to consider limitations arising from variations in skin conditions, such as thickness and color, based on factors like race or individual differences. The optical signal response can vary without accounting for these individual differences in skin conditions. Furthermore, in their study, they employed near-infrared radiation around 2,000 nm, a range where the influence of water absorption cannot be disregarded. Therefore, there is a complexity involved in the alcohol concentration measurement process, requiring correction for the absorption of water in that wavelength range.

Recently, a study on glucose detection using a structure-based Localized Surface Plasmon Resonance (LSPR) sensor was published [27]. In this paper, UV to visible spectrometer, gold nanoparticles (AuNPs), and materials such as the serial quadruple taper fiber (SQTF) and carbon nanotubes were employed to enhance sensitivity for glucose detection. However, the system is relatively complex, and the inconvenience of adding nanoparticles exists. In addition, a study utilizing Surface Plasmon Resonance theory for chemical and biological substance analysis has been reported [28]. However, it has limitations regarding the influence of temperature, humidity, and variations in composition on measurement values. A review paper on biodegradable polymer optical fiber (POF) has been published [29]. Various methods for manufacturing optical fibers for material analysis were discussed, but limitations were reported, such as the susceptibility of optical fibers to breakage, bending, or distortion.

Thus, existing technologies have limitations due to the influence of external factors such as temperature and humidity, requiring periodic calibration, replacement, and facing hygiene concerns. Invasive measurements are reported to cause discomfort and resistance in the measurement subject due to piercing pain, foreign sensations, and the risk of infection [30,31]. Therefore, in this study, a non-invasive alcohol concentration measurement method using optical techniques was investigated to compensate the limitations of existing alcohol measurement methods. 

In this study, we investigated the changes in optical signals with varying concentrations of the alcohol sample solution. By utilizing wavelengths in the near-infrared region, we explored suitable wavelength ranges for future non-invasive blood alcohol concentration measurements. Additionally, we analyzed the optical characteristics of biological substances and aimed to achieve high accuracy through the selection of sensitive data regions. Using liquid samples, we measured changes in optical signals due to alcohol concentration and applied the obtained sensitive wavelength range to mathematical modeling. Finally, we confirmed the applicability of the correlation between optical signals and alcohol concentration for use in an alcohol concentration sensor. The proposed sensor system minimizes the drawbacks associated with the alcohol breathalyzer, particularly the risks of hygiene and infection. Additionally, it eliminates false signals arising from residual alcohol components in the driver’s mouth due to the use of mouthwash or from alcohol present in the air from air fresheners inside the vehicle. The proposed research, following future studies on animal subjects, could be applied to measure human blood alcohol concentration and integrated into a system aimed at preventing drunk driving. In line with the international trend toward legislation on vehicle ignition interlock devices, it is expected to have significant economic ripple effects. Furthermore, it can be applied in various industrial sectors, such as checking whether a surgeon has consumed alcohol before the surgical procedure, determining if heavy equipment operators are under the influence of alcohol before operating machinery, and preventing the operation of dangerous equipment within factories. This can help prevent industrial accidents caused by alcohol consumption.

## 2. Research Background

Near-infrared light has the capability to penetrate deep into the subcutaneous layers of the skin. Therefore, a method for measuring alcohol concentration using the diffuse reflection signals of near-infrared light is proposed. To apply this to the final goal of a button-type ignition interlock device, a reflective measurement method was deemed suitable. Accordingly, in the experiments, the reflective signal measurement method was introduced, utilizing the principles of diffuse reflection. This method detects reflected light signals by leveraging the phenomenon where light signals are absorbed and scattered within the sample before emerging from the sample surface. Through this approach, the reflective signals from the sample are measured to determine the concentration of alcohol.

The experimental data were stored on the PC, and mathematical modeling was carried out using MATLAB 9.14 (R2023a). The experimental setup was constructed using a basic optical table, NIR spectroscopic module, and holder. Through this setup, the feasibility of alcohol concentration measurement using near-infrared was confirmed.

## 3. Materials and Methods

### 3.1. Characteristics of Infrared Light Source

**Figure 1:**
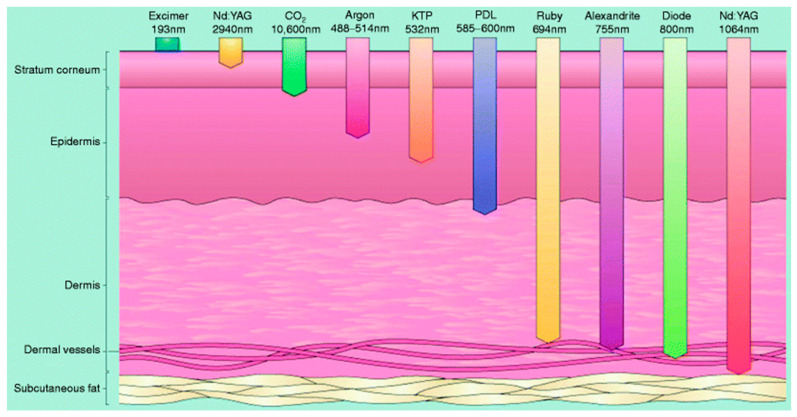
Penetration depth of light energy as a function of wavelength [35].

Electromagnetic spectra are categorized based on the length of wavelengths. As the wavelength increases, it is classified into Gamma rays, X-rays, UV (Ultraviolet), IR (Infrared), and radio waves [32]. Infrared radiation has longer wavelengths than visible light and shorter wavelengths than microwaves. Near-infrared (NIR) light has a wavelength range from 780 nm to 3 μm and can penetrate deep into the skin [33,34]. In this experiment, near-infrared wavelengths that enable the analysis of substances in the subdermal layers of the human body are utilized. Figure 1 illustrates the penetration depth of light energy as a function of wavelength. The visible light region shows penetration depth up to the epidermis, whereas the near-infrared region exhibits penetration depth up to the dermal vessels.

Chemical bonds such as O–H, N–H, and C–H exhibit specific vibrational frequencies in the near-infrared (NIR) wavelength range [36]. When near-infrared (NIR) wavelengths interact with specific substances, the energy of the optical signal is absorbed by the molecules within the substance. Within the near-infrared range, molecular vibrations occur at certain frequencies. When interaction takes place at frequencies matching the molecular vibration frequency, the molecule’s vibration occurs in various forms around its center of mass. In this study, we aim to sense alcohol concentrations by measuring changes in optical signals resulting from these vibrations.

### 3.2. Reflectance and Transmittance Measurement Methods

**Figure 2:**
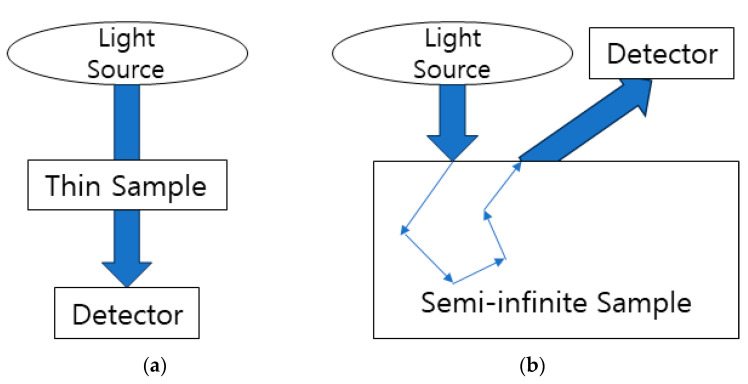
Types of optical signal measurement methods: (**a**) transmittance measurement method; (**b**) reflectance measurement method.

The optical signal measurement methods can be divided into reflectance measurement and transmittance measurement. In the case of transmittance measurement, as shown in Figure 2a, light from the light source passes through the sample and is detected [37]. When the sample is thin and transparent, the measurement signal is significant, but it may not be suitable when the sample is thick or opaque.

Figure 2b illustrates the reflectance measurement method, which utilizes the principle of diffuse reflection [38]. Diffuse reflection is the phenomenon where the optical signal exits the sample surface after being absorbed and scattered within the sample. When the sample thickness is considered semi-infinite, reflected optical signals are not affected by the thickness, making it advantageous [39]. Biological tissues are typically thick and opaque, leading to diffuse reflection, making the reflectance signal measurement method applicable. Therefore, optical energy interacts with a specific substance (e.g., alcohol) through absorption, scattering, and other interactions, and the reflected signal is detected. It can serve as a theoretical basis for measuring the concentration of specific substances.

### 3.3. Sample Preparation

In this experiment, as the first step in alcohol concentration measurement, we conducted the experiment using ethanol solution sample. Ethanol is the main component of alcoholic beverages, and the product used in the experiment (Ethyl Alcohol 99.9% (GR), N4Q202, Duksan Pure Chemicals, Ansan-si, Republic of Korea) has a high ethanol purity of 99.9%.

**Figure 3:**
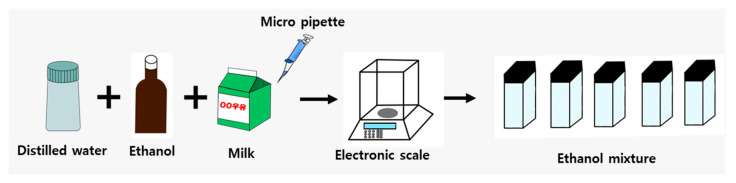
Schematic representation of the sample preparation process for measurements.

The body composition consists of various absorbing and scattering components such as fat, cells, and water. Figure 3 illustrates the sample preparation process. We prepared a total of 10 liquid samples with ethanol concentrations ranging from 0% to 45% in 5% intervals. The human body is treated as a highly scattering medium, and in various studies, milk has been used as a scattering agent [40,41,42,43,44]. To induce scattering effects similar to those in biological samples, we added 10% milk to each sample solution using a micropipette, and the remaining ratio was filled with distilled water. The weights of distilled water (Deionized Water, MF210118681222, Biozoa Biological Supply, Seoul, Republic of Korea), ethanol, and milk, as shown in Figure 3 were measured using an electronic balance and placed in square glass containers with lids.

### 3.4. Sample Measurement Procedures

**Figure 4:**
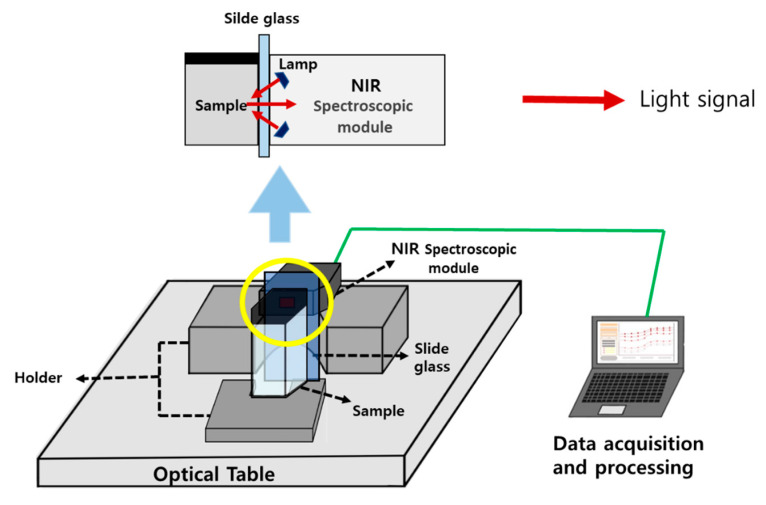
Schematic diagram of the alcohol concentration measurement setup.

Figure 4 illustrates a schematic diagram of the alcohol concentration measurement setup. The alcohol solution sample and the NIR Spectroscopic module (DLP NIRscan Nano EVM, Texas Instruments, Dallas, TX, USA) are aligned and secured with optical holders on the optical table. This module includes a 1.8 mm × 0.025 mm input slit, collimating lenses, an 885 nm long wavepass filter, a reflective diffraction grating, focusing lenses, and collection optics. The center of the liquid sample in the square container is targeted, and a slide glass is positioned to prevent the scratching of the module window and maintain a consistent distance between the module and the sample. The optical signals measured by the module are stored on a PC for later use in data collection, data processing, and mathematical modeling using MATLAB (MathWorks, Natick, MA, USA). We conducted measurements of alcohol samples at different concentrations, scanning each sample 20 times at various wavelengths and derived the average values for each wavelength. This measurement process was repeated a total of 5 times.

**Figure 5:**
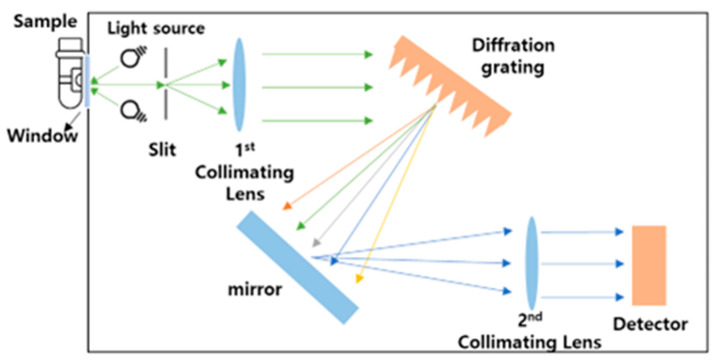
Spectroscopic module schematic.

Figure 5 illustrates the process of optical energy being reflected from the sample and propagated within the spectroscopic module. The wavelength range of the used spectroscopic module is 900 to 1700 nm. Each lamp (light source) emits light at an angle of approximately 40 degrees, designed to focus around 0.75 mm from the sapphire window. The light source concentrates light, creating a spot size that covers the sapphire window. The collimating lens gathers light within a 2.5 mm diameter area from the window. Consequently, the sample must be positioned in direct contact with the sapphire window. The light energy originating from the IR light source is directed through the window onto the sample, where it undergoes diffuse reflection. It then passes through the slit, followed by the first collimating lens, which forms parallel light. Subsequently, the light that encounters the diffraction grating is dispersed according to its wavelength. After being reflected by a micro-mirror, it passes through the second collimating lens and is detected by the detector, resulting in the measurement of optical signals. The absorption and scattering phenomena vary according to the alcohol concentration of the sample. As a result, the diffuse reflection pattern changes, and different optical signals are detected based on wavelength due to dispersion occurring at the diffraction grating.

### 3.5. Data Analysis and Error Minimization

Through the near-infrared spectroscopic module, absorbance data and reflectance data, each consisting of 20 measurements, obtained from samples with different concentrations are saved in Excel format. It was appropriate to apply the inverse transformation method because each dataset increases or decreases monotonically. The inverse transformation, a modeling technique used in this experiment, is the process of converting the relational equation y = f(x) into the form x = f(y). In our data, the x-values correspond to the alcohol concentration, and the y-values correspond to the optical signal, specifically absorbance (Abs). After measuring absorbance at various alcohol concentrations and constructing a graph, we determined the equation that best correlates with the experimental data points; that is, to determine the alcohol concentration by using the change in values of measured data from the optical signals, the obtained data were inversely transformed with first-order and second-order equations, and the fitted equations were created to calculate alcohol concentrations.

**Figure 6:**
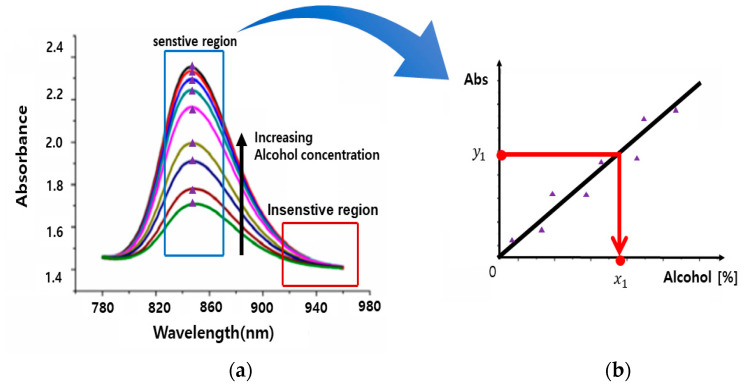
Example of sensitivity region determination and correlation analysis: (**a**) variation of optical signal with wavelength; (**b**) changes in optical signal with alcohol concentration at the optimal sensitive wavelength.

When examining the graph as shown in Figure 6a, it is evident that there are sensitive regions where the difference in optical signals distinguishes alcohol concentrations clearly, as well as insensitive regions where this difference is less noticeable. To determine the wavelength range with high optical signal sensitivity, a curve fitting using polynomial interpolation is performed in MATLAB. As depicted in Figure 6b, the optimal wavelength within the sensitive range is selected, and a graph of optical signal vs. alcohol concentration is plotted at that wavelength. This allows for the calculation of the corresponding alcohol concentration based on the measured optical signal.

**Figure 7:**
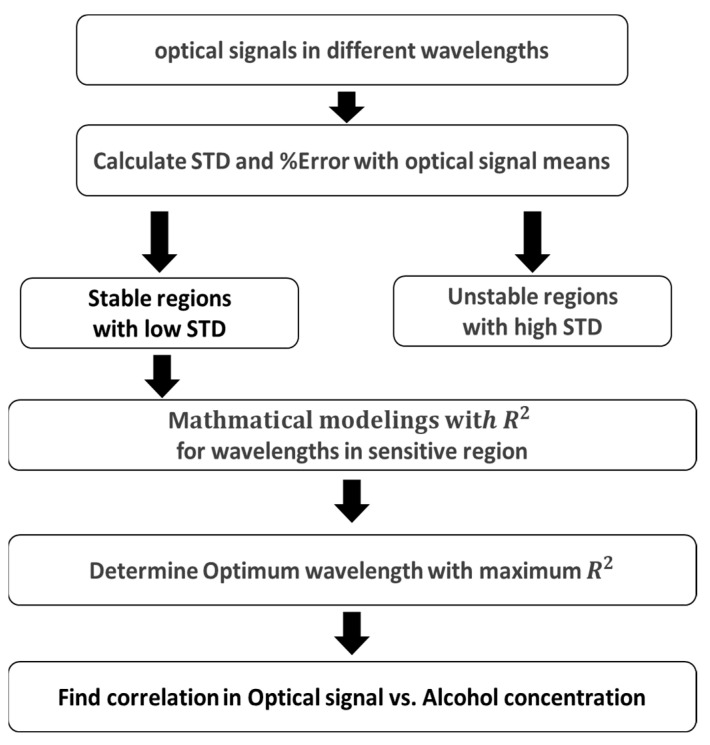
Schematic representation of overall data processing and analysis.

Figure 7 illustrates the schematic representation of the overall data processing and analysis. After obtaining optical signal values at various wavelength ranges, the mean and standard deviation (STD) are calculated for each. Stable and unstable wavelength regions are identified in the graphical representation of optical signal vs. wavelength. Mathematical modeling is performed on the stable wavelength range to obtain first- and second-degree equations for each wavelength along with their coefficient of determination (*R*^2^) values. These *R*^2^ values at each wavelength are compared to select the optimal wavelength. The correlation between the selected optimal wavelength and alcohol concentration is then determined.

## 4. Results and Discussion

### 4.1. Optical Signals as a Function of Wavelength: Determination of Stable, Unstable, and Sensitive Region

**Figure 8:**
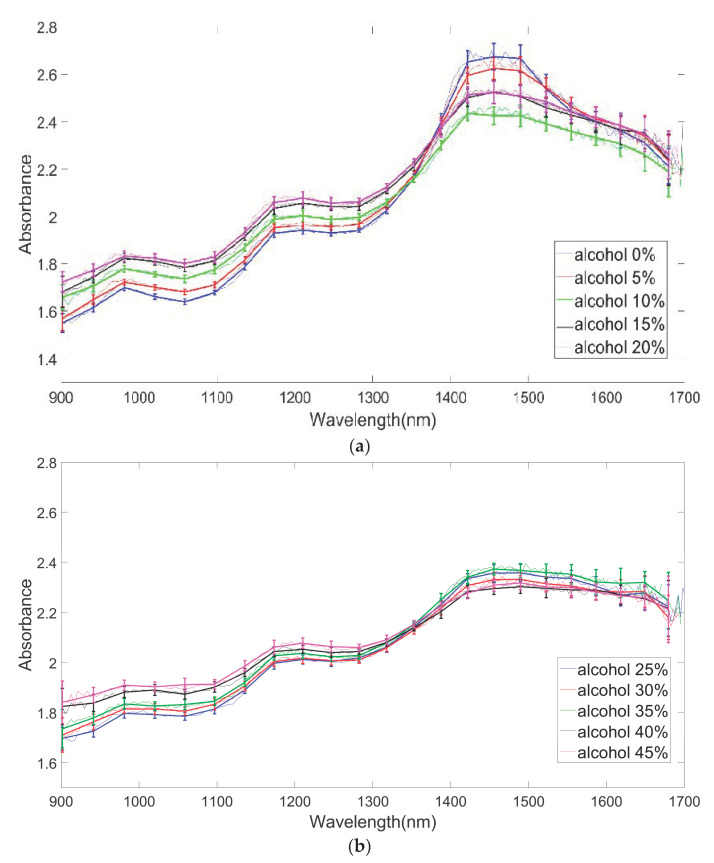
Changes in optical signal with respect to wavelength: (**a**) alcohol concentration ranging from 0% to 20%; (**b**) alcohol concentration ranging from 25% to 45%.

Figure 8 illustrates the variation in measured optical signals as a function of wavelength. To mimic the scattering characteristics of biological tissue, 10 mL of milk was added as scatterer, and experiments were conducted at various alcohol concentrations. Figure 8a displays the optical signals (absorbance) as a function of wavelength in the alcohol concentration range from 0 to 20%. Figure 8b shows the optical signals (absorbance) as a function of wavelength in the alcohol concentration range from 25% to 45%.

**Figure 9:**
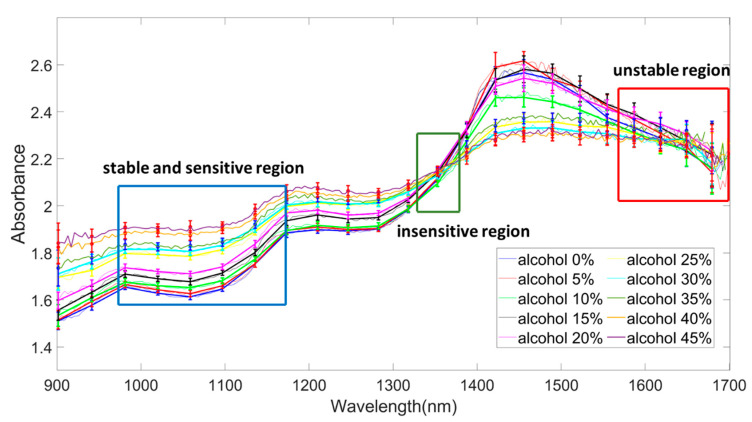
Stable region, unstable region, and sensitive region of optical signal with respect to wavelength.

Figure 9 illustrates the absorbance changes as a function of wavelength across the entire range of alcohol concentrations from 0% to 45%. Areas with relatively large standard deviations were defined as the unstable region, while areas with low standard deviations were designated as the stable region. Furthermore, we have designated the region with unclear differentiation between concentrations as the insensitive region. A stable and sensitive region was selected, and mathematical modeling was performed at each wavelength within that region. By examining Figure 9, it is evident that there is an unstable region where the standard deviation of data is high, noise is prevalent, and the data exhibit inversion concerning alcohol concentration vs. optical signal. In contrast, there is also a stable region characterized by low standard deviation, minimal noise, and no data inversion.

Table 1 presents the percent error values for alcohol concentration in the stable region (wavelength range 1032.64~1067.45 nm) and unstable region (wavelength range 1627.161~1655.038 nm). The stable region was determined based on relatively small percent error values. Table 1a shows the %Error with respect to wavelength for alcohol concentrations ranging from 0% to 20%. Table 1b displays the %Error with respect to wavelength for alcohol concentrations ranging from 25% to 45%. The data from the stable region were later used for modeling the correlation between changes in optical signals and alcohol concentration. Upon reviewing Table 1, it is evident that in the stable region, %Error values range from approximately 0.67% to 1.25%, while in the unstable region, %Error values are around 3.5%. Based on the calculated %Error values, it is observed that the %Error values in the unstable region are approximately 3 to 3.5 times larger than those in the stable region. Therefore, based on these results, the wavelength ranges from 1032.64 nm to 1067.45 nm, which exhibited high reliability, was selected as the stable and sensitive region.

### 4.2. Alcohol Concentration Sensing through Mathematical Modeling

Table 2 shows the average values of absorbance within the alcohol concentration range of 0 to 45%. Additionally, it provides the R-squared values and *p*-values for the mathematical modeling results of both the first-order and second-order equations at each wavelength within the stable region. Table 2a corresponds to the wavelength range of 1032.64 to 1047.6 nm. Table 2b corresponds to the wavelength range of 1051.33 to 1067.45 nm. As shown in Table 2, at the wavelength of 1051.v33 nm, the R-squared value for the first-order equation is 0.9908, and the R-squared value for the second-order equation is 0.9938, with *p* < 0.001. Therefore, the wavelength of 1051.33 nm exhibits the highest R-squared values for both the first-order and second-order equations compared to other wavelengths, indicating it as the optimal wavelength.

**Figure 10:**
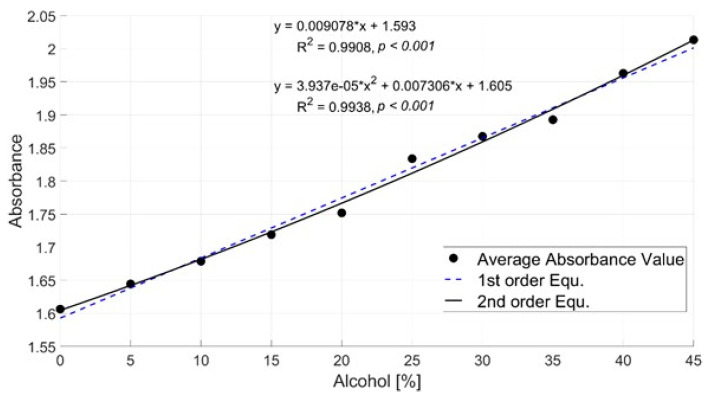
The variation in optical signals with respect to alcohol concentration.

Figure 10 shows the graph depicting the changes in optical signal (absorbance) with respect to alcohol concentration at the selected sensitive wavelength of 1051.33 nm. Modeling was performed using both first-order and second-order equations for alcohol concentrations ranging from 0% to 45%. The R-squared value for the first-order equation is 0.9908, while the R-squared value for the second-order equation is 0.9938. Furthermore, the *p*-value is less than 0.001, confirming statistical significance.

The purpose of this experiment is to investigate the changes in optical signals based on alcohol concentration, aiming to study the correlation between optical signals and alcohol concentration using near-infrared spectroscopy (NIR) for alcohol concentration measurement. To achieve this, we fitted the optical signal values concerning alcohol concentration to graphs. Figure 10 shows the absorbance graph of alcohol concentration vs. optical signal values at a high confidence wavelength. Upon examination of the graph, both the first-order equation with an R-squared value of 0.9908 and the second-order equation with an R-squared value of 0.9938 exhibit high correlations. When inspecting the data points, it is evident that they demonstrate an increase in optical signal values as the concentration increases. As demonstrated in the modeling equations from Figure 10, the R-squared values for the first-order equation (0.9908) and the second-order equation (0.9938) do not differ significantly. Therefore, it has been confirmed that utilizing the results from the first-order equation modeling to calculate alcohol concentration will yield similar accuracy, allowing for a simpler modeling approach.

The ultimate goal of this study is to develop a non-invasive optical spectroscopy technique for measuring human alcohol concentration and apply it to a system for preventing drunk driving. This study, as the first step toward achieving the ultimate goal, is an in vitro investigation measuring alcohol concentration in liquid samples. However, it has the following limitations. The human body consists of skin layers and subcutaneous layers, with blood vessels primarily distributed in the subcutaneous layer. To analyze alcohol concentration inside the body using optical signals, research on the changes in optical signals due to the skin is essential. Additionally, the internal environment of the body contains a mixture of various substances besides alcohol. For a single sample conducted in this study, the %Error at the optimal wavelength for each concentration ranged from 0.7% to 1.2%, and the reproducibility was confirmed with an R-squared value of 0.9908 for the first-order equation and 0.9938 for the second-order equation. However, reproducibility and stability have not yet been verified in complex samples containing mixtures of glucose, fats, hemoglobin, and other components. Thus, it is necessary to correct for changes in optical signals due to other components such as proteins, fats, glucose, etc.

Therefore, in the next stage of research, additional studies based on skin thickness and color will be conducted. Moreover, when dealing with mixtures of various substances within the body, the optical signals may be influenced by components other than alcohol. Hence, in future research, mathematical modeling and signal processing will be undertaken to extract only the alcohol signals from such complex mixtures. Despite the mentioned limitations, the significance of this study lies in conducting a foundational research step toward implementing a system for preventing road traffic accidents caused by drunk driving. This research confirms the potential for such a system, serving as the first step in its development. It demonstrates the feasibility of analyzing alcohol components in a solution using non-invasive optical methods for possible application in a button-type ignition interlock device to prevent drunk driving.

## 5. Conclusions

In this study, we implemented a non-invasive optical technique for measuring alcohol concentration. A reflectance measurement method was used, which can potentially be applied to measure blood alcohol concentration non-invasively. This method could be integrated into anti-drunk driving systems, using non-invasive reflective sensors on components like the ignition button, to detect the driver’s alcohol state and prevent the vehicle from starting if necessary. We employed a module operating in the near-infrared wavelength range with relatively deep tissue penetration to determine stable, unstable, and sensitive regions of the optical signal based on wavelength. Using the wavelengths within the sensitive region, we established correlation equations between the optical signal and alcohol concentration using first-order and second-order equations. Subsequently, we selected the wavelength with the highest *R*^2^ values as the optimal wavelength. Since there is no significant difference between the values of the first-order and second-order equations, we demonstrated that using the first-order modeling equation at the optimal wavelength allows for the precise sensing of alcohol concentration in liquid samples.

This study investigated changes in optical absorbance across a wide range of alcohol concentrations to explore the feasibility of alcohol concentration measurement using optical methods. To apply the findings to practical alcohol measurement scenarios, further research within the legally defined alcohol concentration ranges is necessary. Therefore, we plan to conduct future studies involving biologically relevant substances, animal experiments, and human subjects, incorporating legally specified alcohol concentration levels. The suggested study, once validated through subsequent animal trials, can be applied to measure blood alcohol concentration in humans, making it applicable for integration into drunk driving prevention systems. Furthermore, it can find application in various industries, such as determining the sobriety of surgeons in hospital operating rooms, preventing alcohol-impaired heavy machinery operation, and averting industrial accidents caused by alcohol consumption in factories. If applied in the future to a non-invasive ignition button-based alcohol prevention system, this study is expected to have significant economic and social implications. 

## Figures and Tables

**Table 1:** %Error values of optical signals according to wavelength and alcohol concentration: (a) %Error values in the range of 0% to 20%; (b) %Error values in the range of 25% to 45%.

(a)						
The %Error by Alcohol Concentration is calculated as follows: %Error = (STD/Absorbance Mean Value) ∗ 100						
	Concentration	0%	5%	10%	15%	20%
Wavelength						
**1032.64 nm to 1067.45 nm (Stable Region)**						
1032.64 nm		0.71%	0.55%	0.69%	0.80%	0.51%
1036.39 nm		0.70%	0.57%	0.64%	0.57%	0.62%
1040.13 nm		0.65%	0.65%	0.52%	0.82%	0.58%
1043.87 nm		0.50%	0.65%	0.63%	0.77%	0.74%
1047.6 nm		0.42%	0.70%	0.63%	0.64%	0.77%
1051.33 nm		0.58%	0.66%	0.81%	0.71%	0.74%
1055.06 nm		0.51%	0.71%	0.68%	0.76%	0.71%
1058.78 nm		0.72%	0.80%	0.50%	0.81%	0.62%
1062.5 nm		0.69%	0.72%	0.57%	0.67%	0.70%
1067.45 nm		0.65%	0.92%	0.54%	0.77%	0.73%
**1627.161 nm to 1655.038 nm (Unstable Region)**						
1627.161 nm		2.06%	2.44%	1.67%	2.55%	2.80%
1630.166 nm		1.37%	1.77%	1.81%	2.17%	1.89%
1634.165 nm		2.11%	2.16%	2.86%	2.09%	2.43%
1637.159 nm		2.19%	2.53%	2.32%	1.90%	2.38%
1640.15 nm		1.71%	2.50%	2.22%	2.47%	2.50%
1643.136 nm		3.35%	3.11%	2.18%	2.62%	2.70%
1646.118 nm		2.64%	2.54%	1.86%	2.01%	3.08%
1649.095 nm		2.80%	2.08%	2.96%	1.93%	2.24%
1652.069 nm		3.25%	3.29%	2.15%	2.14%	1.76%
1655.038 nm		3.04%	2.45%	2.91%	2.94%	2.38%
**(b)**						
**The %Error by Alcohol Concentration is calculated as follows: %Error = (STD/Absorbance Mean Value) ∗ 100**						
	**Concentration**	**25%**	**30%**	**35%**	**40%**	**45%**
**Wavelength**						
**1032.64 nm to 1067.45 nm (Stable Region)**						
1032.64 nm		0.87%	1.12%	0.94%	0.98%	1.98%
1036.39 nm		1.08%	1.05%	1.08%	0.80%	1.68%
1040.13 nm		0.68%	1.19%	1.27%	1.55%	1.12%
1043.87 nm		0.91%	1.40%	1.00%	1.13%	1.30%
1047.6 nm		1.22%	1.10%	1.00%	1.31%	1.51%
1051.33 nm		1.06%	1.60%	1.21%	1.29%	1.21%
1055.06 nm		1.20%	1.21%	1.09%	1.63%	1.68%
1058.78 nm		1.18%	1.17%	1.13%	1.72%	1.90%
1062.5 nm		1.12%	1.33%	1.42%	1.09%	1.86%
1067.45 nm		1.05%	1.23%	1.42%	1.33%	1.43%
**1627.161 nm to 1655.038 nm (Unstable Region)**						
1627.161 nm		3.12%	4.42%	3.47%	4.69%	6.66%
1630.166 nm		1.98%	4.54%	3.40%	4.48%	4.33%
1634.165 nm		3.61%	4.16%	3.41%	2.89%	5.14%
1637.159 nm		2.99%	4.33%	3.42%	3.15%	7.73%
1640.15 nm		5.69%	4.05%	3.16%	5.90%	4.69%
1643.136 nm		3.78%	3.69%	3.66%	3.83%	6.75%
1646.118 nm		4.75%	2.83%	4.44%	4.77%	5.03%
1649.095 nm		3.48%	4.92%	2.65%	4.60%	4.87%
1652.069 nm		2.53%	4.46%	4.78%	4.52%	6.76%
1655.038 nm		3.76%	3.81%	6.31%	4.04%	4.84%

**Table 2:** Absorbance average, R-squared (*R*^2^), and *p*-values for each wavelength in the stable region: (a) values between 1032.64 nm and 1047.6 nm; (b) values between 1051.33 nm and 1067.45 nm.

(a)						
	Wavelength	1032.64 nm	1036.39 nm	1040.13 nm	1043.87 nm	1047.6 nm
Concentration						
**1032.64 nm to 1047.6 nm**						
0%		1.61387	1.61216	1.60997	1.60964	1.60796
5%		1.65314	1.64823	1.67993	1.72509	1.75513
10%		1.685	1.67993	1.67992	1.67967	1.68234
15%		1.72717	1.72509	1.72367	1.72231	1.7199
20%		1.75906	1.75513	1.75436	1.75482	1.74988
25%		1.84298	1.84295	1.84251	1.83839	1.83809
30%		1.87355	1.87141	1.87539	1.87255	1.86992
35%		1.89596	1.89496	1.89522	1.8937	1.89469
40%		1.97608	1.96561	1.9639	1.97283	1.96597
45%		2.02508	2.01502	2.01193	2.01407	2.01243
*R* ^2^	1st-order Equ.	0.9884	0.9893	0.9897	0.9895	0.9897
	2nd-order Equ.	0.9922	0.9918	0.9914	0.9923	0.9923
*p-value*		<0.001 ***	<0.001 ***	<0.001 ***	<0.001 ***	<0.001 ***
**(b)**						
	**Wavelength**	**1051.33 nm**	**1055.06 nm**	**1058.78 nm**	**1062.5 nm**	**1067.45 nm**
**Concentration**						
**1051.33 nm to 1067.45 nm**						
0%		1.6064	1.60737	1.60746	1.61086	1.60862
5%		1.64452	1.64504	1.66594	1.6481	1.64808
10%		1.67878	1.67861	1.67762	1.68021	1.68566
15%		1.71902	1.71943	1.71904	1.72162	1.72089
20%		1.75184	1.7518	1.75159	1.75364	1.75488
25%		1.83386	1.83811	1.8399	1.84132	1.83963
30%		1.86756	1.86963	1.86946	1.87028	1.8696
35%		1.89271	1.89321	1.89781	1.891	1.8965
40%		1.96288	1.96401	1.96687	1.96939	1.9668
45%		2.01344	2.0106	2.01733	2.01363	2.01351
*R* ^2^	1st-order Equ..	0.9908	0.9901	0.9898	0.9881	0.9908
	2nd-order Equ.	0.9938	0.9924	0.9928	0.991	0.9932
*p-value*		<0.001 ***	<0.001 ***	<0.001 ***	<0.001 ***	<0.001 ***

* *p* < 0.05, ** *p* < 0.01, *** *p* < 0.001.

## Data Availability

Data are contained within the article.
